# Optimization of Malachite Green Removal from Water by TiO_2_ Nanoparticles under UV Irradiation

**DOI:** 10.3390/nano8060428

**Published:** 2018-06-13

**Authors:** Yongmei Ma, Maofei Ni, Siyue Li

**Affiliations:** 1 Chongqing Institute of Green and Intelligent Technology, Chinese Academy of Sciences, Chongqing 400714, China; mayongmei@cigit.ac.cn (Y.M.); nimaofei000@163.com (M.N.); 2 University of Chinese Academy of Sciences, Beijing 100049, China

**Keywords:** TiO_2_, photocatalytic degradation, malachite green, kinetics process, concentration gradient

## Abstract

TiO_2_ nanoparticles with surface porosity were prepared by a simple and efficient method and presented for the removal of malachite green (MG), a representative organic pollutant, from aqueous solution. Photocatalytic degradation experiments were systematically conducted to investigate the influence of TiO_2_ dosage, pH value, and initial concentrations of MG. The kinetics of the reaction were monitored via UV spectroscopy and the kinetic process can be well predicted by the pseudo first-order model. The rate constants of the reaction kinetics were found to decrease as the initial MG concentration increased; increased via elevated pH value at a certain amount of TiO_2_ dosage. The maximum efficiency of photocatalytic degradation was obtained when the TiO_2_ dosage, pH value and initial concentrations of MG were 0.6 g/L, 8 and 10^−5^ mol/L (M), respectively. Results from this study provide a novel optimization and an efficient strategy for water pollutant treatment.

## 1. Introduction

Environmental water pollution is becoming more serious with the development of the social economy and the high density of industrial activity [1,2]. Many countries have suffered water pollution because of the indiscriminate release of untreated wastewater. Among all water, environmental contaminants, synthetic dye, a typical organic pollutant, such as malachite green (MG, C_23_H_25_CN_2_), has been attracting growing attention [3,4,5]. MG is a common triphenylmethane dye in the textile industry, and it has also been widely used in the fish farming industry as a fungicide, disinfectant, ectoparasiticide, and antibacterial agent [6,7]. However, many researchers have reported that MG and its metabolite leucomalachite green (LMG) are environmentally persistent due to their complex chemical structures. They may also lead to teratogenic, carcinogenic, and mutagenic effects in human beings [8,9]. Thus, MG has been banned or is restricted in many countries. Nevertheless, MG is still illegally used in aquaculture because of its high efficacy and low cost [10]. To minimize the harm caused by MG, it is very important to efficiently remove MG residue from water systems.

In the past few decades, several methods have been applied for MG removal from wastewater. The conventional methods used for MG removal include adsorption, biodegradation, oxidation with ozone or hydrogen peroxide, membrane technology, etc. [11,12,13]. However, many of these methods are costly, time-consuming, difficult to control, and have high energy consumption and low efficiency. Fortunately, an alternative to the methods mentioned above is advanced oxidation processes, of which photocatalysis is the most popular. The photocatalytic degradation of pollutants through the use of nanomaterials has aroused world-wide attention. In the photocatalysis process, a semiconductor oxide is needed to generate radicals under illuminated light, which are the responsible active species for removal of hazardous compounds [14]. Among the various photocatalyst, titanium dioxide (TiO_2_) has been widely used in degrading organic pollutants due to its strong oxidizing power under ultraviolet (UV) light, extraordinary chemical stability, biocompatible features, relatively low cost, and environmental friendliness [15,16,17]. The principle of TiO_2_ photocatalytic properties is straightforward: When TiO_2_ absorbs energy greater than the band gap (approximately 3.2 eV) of itself, electrons can be excited to create electron-hole pairs. These electron-hole pairs migrate to the surface and form hydroxyl groups, which can react with chemicals adsorbed there [18,19]. Therefore, simple, low-cost, and high-efficiency TiO_2_ used as a photocatalyst to degrade pollutants is considered an attractive and promising treatment for water pollution.

Herein, we report a simple hydrothermal method to prepare TiO_2_ as an efficient photocatalyst for MG treatment from water under irradiation of UV light. Our procedure for material fabrication is easily operated, has a low cost and is scalable. We tried to determine the optimum operation conditions that can improve the efficiency of MG removal. A series of contrast experiments were performed to confirm TiO_2_ dosage, pH, and the concentrations of MG, which can maximize the utilization efficiency of TiO_2_ under UV lights. The whole dynamic process of photocatalytic degradation of MG with TiO_2_ under UV lights was monitored using a UV-vis spectrophotometer to evaluate the efficiency of MG removal. The results revealed that this simple photocatalytic platform can efficiently remove water pollutants.

## 2. Results and Discussion

### 2.1. Characterization of TiO_2_

**Figure 1:**
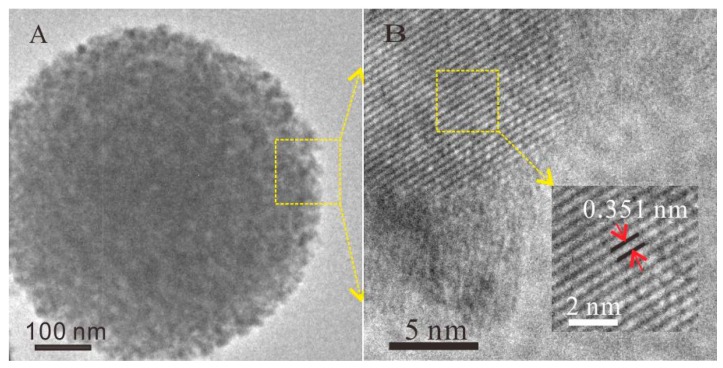
(**A**) TEM image of the TiO_2_ particle; (**B**) enlarged HRTEM image of details of the TiO_2_ particle.

TiO_2_ particles were prepared according to a classical hydrothermal method [19]. Transmission electron microscopy (TEM) and scanning electron microscopy (SEM) images demonstrated that the TiO_2_ particles were sphere-shaped and had a uniform size distribution, with a diameter around 600 nm (Figure 1 and Figure S1). High resolution transmission electron microscopy (HRTEM) images demonstrated that the lattice fringe, with a spacing of about 0.35 nm, corresponded to the (101) plane of anatase titania. It also revealed that the TiO_2_ particles were well-dispersed without any aggregation, indicating the stability of these dispersions. The UV-vis spectra showed that TiO_2_ had an absorbance peak centered at 249 nm (Figure S2).

**Figure 2:**
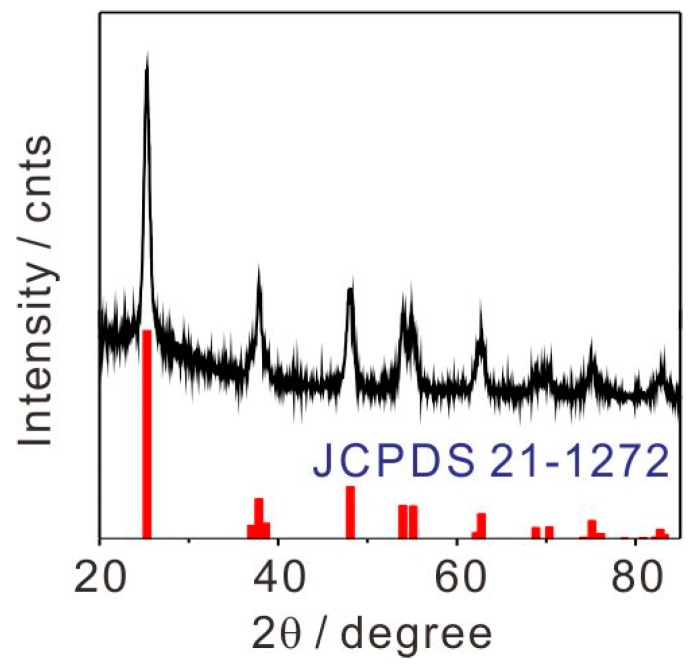
XRD patterns of TiO_2_ and the standard XRD patterns of anatase-phase TiO_2_ (JCPDS 21-1272).

The X-ray diffractometer (XRD) pattern of the TiO_2_ nanoparticles (Figure 2) were in agreement with the standard values of anatase-phase TiO_2_, with peaks at 2θ = 25.3°, 38.6°, 48.1°, 54.3°, 55.4°, 62.8°, 68.9°, 70.4°, 75.2°, 83.2° (JCPDS files No. 21-1272). Anatase-phase TiO_2_ has been reported to demonstrate the best photocatalytic degradation activity among various TiO_2_ crystallinities [20].

### 2.2. Kinetics of Photocatalytic Degradation

#### 2.2.1. TiO_2_ Dosage Effect on MG Removal

MG and UV light were used to investigate the photocatalytic activity of TiO_2_ particles. TiO_2_ particles were dispersed in MG solution (100 mL) in a quartz tube. For obtaining the accurate concentration of MG in photocatalytic degradation, the solution was first stirred for 2 h in the dark to reach an adsorption–desorption equilibrium between the nanoparticles and the solution. Upon UV light irradiation for a designated time, 3 mL of MG aqueous solution was taken out and centrifuged, then the supernatant was used for measuring the absorbance by UV-vis spectroscopy. In practical systems, the optimal operating conditions are very important for the efficiency of pollutant removal [21]. Thus, the optimal amount of TiO_2_, pH value and the concentrations of MG would be obtained in the next series of experiments.

**Figure 3:**
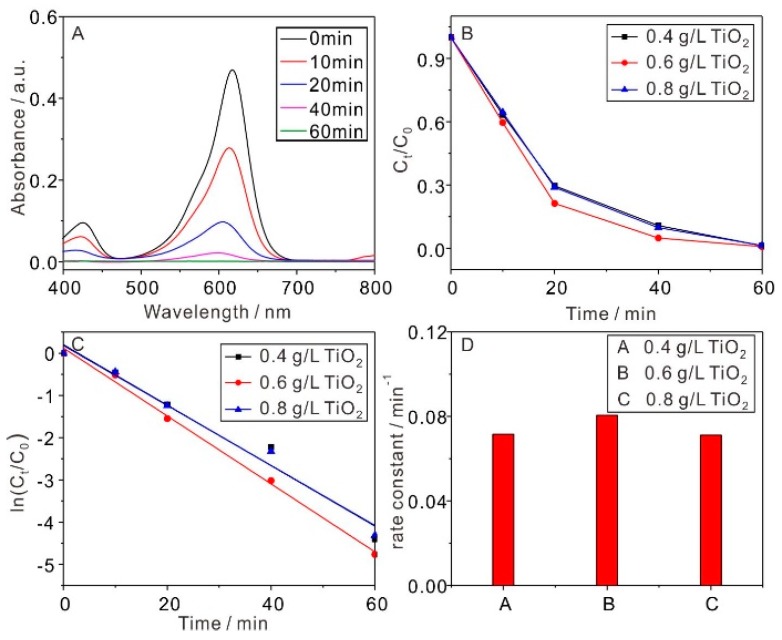
(**A**) Time-course UV-Vis absorbance spectra of MG (10^−5^ M) photocatalyzed by 0.6 g/L TiO_2_ under UV light; (**B**–**D**) The calculated time-dependent ratios of *C_t_*/*C*_0_, first-order degradation rates, and reaction rate constants under UV light with different TiO_2_ dosage.

Figure 3 demonstrates the influence of amounts of TiO_2_ on the efficiency of MG removal. The absorbance spectra of MG (1 × 10^−5^ M) photocatalyzed by 0.6 g/L TiO_2_ particles under UV light irradiation, as a function of irradiation time, are shown in Figure 3A. With the increasing of irradiation time, the absorption intensity of MG at 615 nm gradually decreased, indicating the degradation of MG under the used conditions. The photocatalytic activity of TiO_2_ could be evaluated through a comparison between the supernatant concentration at each exposure time to that at time zero. Figure 3B plots time-dependent curves of the *C_t_*/*C*_0_ ratio, an indicator of the degradation degree. *C*_0_ was the original concentration of MG at time zero and *C_t_* was the supernatant concentration of MG after the sample was photocatalyzed by TiO_2_ particles under UV light for time *t*. *C_t_* was obtained from comparing the 615 nm absorbance of the MG supernatant with that of the standard MG solution. After 1 h of irradiation, almost all the MG solution was photocatalytic degraded, regardless of the amount of TiO_2_. However, the degradation rates of the different amounts of TiO_2_ were different during the whole photocatalytic degradation process. In order to further evaluate the degradation rates mentioned above, Figure 3C shows the kinetics of the photocatalytic degradation reactions, which can be described as a pseudo first-order by Equation (1) [22].

(1) $$\ln\frac{C_{t}}{C_{0}}=-kt$$

The rate constants (*k*, min^−1^) were calculated from plots of ln (*C_t_*/*C*_0_) vs. irradiation time. The calculated rate constants with 0.4, 0.6 and 0.8 g/L of TiO_2_ are 0.0716, 0.0805, and 0.0711 min^−1^, respectively (Figure 3D and Table 1). The results indicated that the rate constant reached best when TiO_2_ dosage was 0.6 g/L under current conditions. The reaction rate constant was found to decrease with increasing TiO_2_ dosage up to 0.8 g/L. The probable reason is that light scattering induced by the increased turbidity can reduce the UV light penetration into the bulk suspension and counteract the effect of photocatalyst surface area, resulting in decreased efficiency of MG removal [23,24]. The *R*^2^ of pseudo first-order kinetic model for the photocatalytic degradation of MG with 0.4, 0.6 and 0.8 g/L of TiO_2_ are 0.964, 0.995, and 0.976, respectively (Table 1). The *R*^2^ values indicated that there was a better correlation to photocatalytic degradation of MG under TiO_2_ particles based on the pseudo first-order kinetic model [25].

#### 2.2.2. Initial Concentrations of MG Effect on the Efficiency of Photocatalytic Degradation

**Figure 4:**
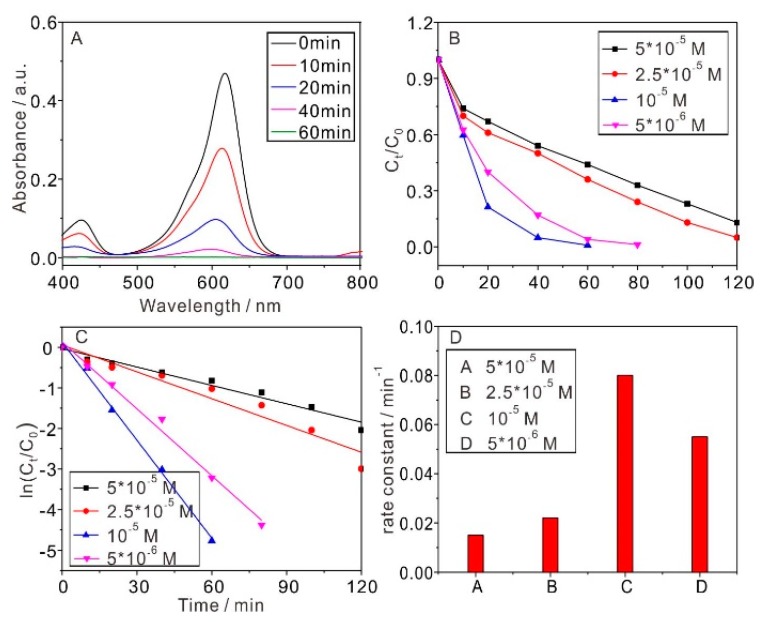
(**A**) Time-course UV-vis absorbance spectra of MG (10^−5^ M) photocatalyzed by 0.6 g/L TiO_2_ under UV light; (**B**–**D**) The calculated time-dependent ratios of *C_t_*/*C*_0_, first-order degradation rates, and reaction rate constants for different concentration of MG photocatalyzed by 0.6 g/L TiO_2_ under UV light.

To obtain the capacity of the photocatalyst, different concentrations of MG solution were photocatalyzed using 0.6 g/L TiO_2_ under UV light (Figure 4). Figure 4A shows the absorbance spectra of MG (1 × 10^−5^ M) photocatalyzed using 0.6 g/L TiO_2_ particles under UV light irradiation as a function of the irradiation time. Similarly, Figure 4B plots time-dependent curves of the *C_t_*/*C*_0_ ratio to indicate the photocatalytic activity of TiO_2_. After 1 h irradiation, almost all MG solution with concentrations of 10^−5^ and 5 × 10^−6^ M had been photocatalytically degraded. Fifty-six percent of the MG solution (5 × 10^−5^ M) was degraded, and 64% of MG solution (2.5 × 10^−5^ M) was degraded after 1 h. Meanwhile, both of the degradation ratios of MG increased continuously with growing irradiation time and reached about 99% after 2 h of irradiation.

The kinetics of the photocatalytic degradation reactions also can be described as pseudo first-order according to Equation (1) (Figure 4C). The calculated rate constants with the MG concentrations of 5 × 10^−5^, 2.5 × 10^−5^, 10^−5^ and 5 × 10^−6^ M were 0.0151, 0.0220, 0.0805 and 0.0550 min^−1^, respectively (Figure 4D and Table 2), using the methods mentioned above. The results demonstrated that the rate constant reached its best performance when the concentration of MG was 10^−5^ M under the used conditions. Generally, the reaction rate constant will increase by decreasing the concentration of pollutants. However, the ability of the photocatalyst may not show efficiently when the concentration of pollutants is too low. Thus, 10^−5^ M of MG was chosen in subsequent experiments. The *R*^2^ of the pseudo first-order kinetic model for the photocatalytic degradation of MG, with concentrations of 5 × 10^−5^, 2.5 × 10^−5^, 10^−5^ and 5 × 10^−6^ M, were 0.965, 0.935, 0.995 and 0.989, respectively (Table 2). The results indicated that the correlation to photocatalytic degradation of MG under TiO_2_ particles based on the pseudo first-order kinetic model was good.

#### 2.2.3. Effect of pH Values

**Figure 5:**
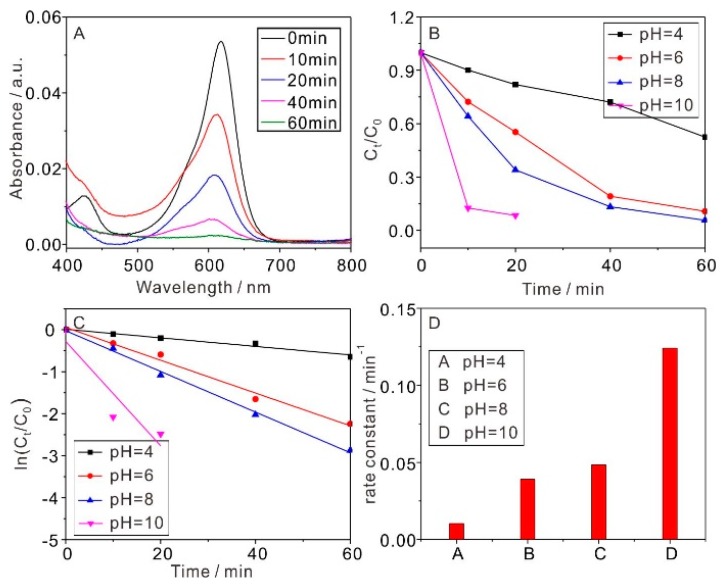
(**A**) Time-course UV-Vis absorbance spectra of MG (10^−5^ M) photocatalyzed by 0.6 g/L TiO_2_ under UV light at pH = 8. (**B**–**D**) The calculated time-dependent ratios of *C_t_*/*C*_0_, first-order degradation rates and reaction rate constants for MG (10^−5^ M) photocatalyzed by 0.6 g/L TiO_2_ with different pH values.

The pH of the aqueous solution is a significant parameter which influences the efficiency of the photocatalytic degradation at the solution-photocatalyst interfaces [26]. Figure 5 indicates the influence of pH on the efficiency of photocatalytic degradation. The absorbance spectra of MG (1 × 10^−5^ M) photocatalyzed by 0.6 g/L TiO_2_ particles at pH = 8 under UV light irradiation, as a function of the irradiation time, are shown in Figure 5A. The time-dependent curves of the *C_t_*/*C*_0_ ratio were also used to indicate the photocatalytic activity of TiO_2_ (Figure 5B)_._ After 1 h of irradiation, 48%, 90% and 95% of MG solution were degraded at pH = 4, 6 and 8, respectively. When the pH value was 10, the degradation ratio of MG reached about 92% after only 20 min of irradiation.

The kinetics of photocatalytic degradation was determined using Equation (1), and the reaction rate constant could be readily derived from the linearly-fitted slope (Figure 5C). The calculated rate constants at pH = 4, 6, 8 and 10 were 0.010, 0.039, 0.048 and 0.124 min^−1^, respectively (Figure 5D and Table 3). The results indicated that the reaction rate constant increased with the increasing of the pH values. The surface of the photocatalyst acquired a positive charge when the pH of the solution was less than 7. The amount of MG on the surface of the photocatalyst decreases because of the electrostatic repulsion between the positive surface of photocatalyst and the positive surface of MG. Meanwhile, a photocatalytic degradation reaction generally occurs on the surface of a photocatalyst [27,28]. Thus, acidic conditions were a disadvantage for the reaction. On the contrary, alkaline conditions can promote MG molecules to the surface of the photocatalyst because of the electrostatic attractions between the negative surface of the photocatalyst and the positive surface of MG, resulting in a high efficiency of reaction. The *R*^2^ of pseudo first-order kinetic models for the photocatalytic degradation of MG at pH = 4, 6, 8 and 10 were 0.961, 0.983, 0.995 and 0.737, respectively (Table 3). The results indicated that the correlation to the photocatalytic degradation of MG under TiO_2_ particles based on the pseudo first-order kinetic model was good, except for when the pH value was 10. In general, water is weak to mildly alkaline in nature, pH = 8 is close to the pH of natural riverine water. Thus, this condition has great practical application potential in photocatalytic treatment. More importantly, the efficiency of reaction at pH = 8 was also good. Overall, the relatively moderate conditions of pH = 8 were better for this reaction.

### 2.3. Mechanism of Photocatalytic Degradation

**Scheme 1:**
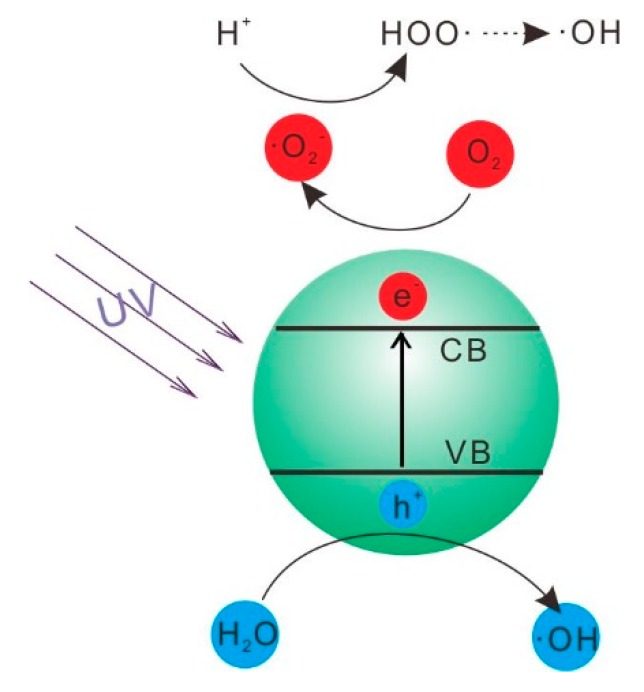
Illustrated mechanism of UV-activated photocatalysis on TiO_2_.

In order to understand the possible mechanisms for the UV-activated photocatalytic degradation activity of TiO_2_ nanoparticles, electron spin resonance (ESR) measurements were conducted. The spectra displaying signals with the characteristic intensity 1:2:2:1 for DMPO-·OH adducts was obtained, which indicated that the ·OH radical was formed under the used conditions (Figure S3) [29]. The possible mechanism for the photocatalytic degradation reaction was discussed (Scheme 1). Under the irradiation of UV light, TiO_2_ absorption the energy larger than its band gap (approximately 3.2 eV), electrons were excited from the valence band (CB) to the conduction band (VB), creating electron-hole pairs [30]. And then these electron−hole pairs will migrate to the surface and take part in surface reactions. When the excited electrons arrived at the surface, they reacted with the oxygen to form superoxide ·O^2−^ radical anions. The superoxide ·O^2−^ radical anions reacted with H^+^, and finally formed hydroxyl radicals (·OH). Meanwhile, holes also reacted with H_2_O and formed ·OH [15,31]. Therefore, the organic molecules present in the solution could react with these oxidizing agents to induce oxidative degradation. In addition, the strong oxidizing power of ·OH could oxidize most of the organics to carbon dioxide (CO_2_) and water (H_2_O).

## 3. Materials and Methods

### 3.1. Chemicals and Materials

Titanium n-butoxide (Ti(OBu)_4_), alcohol and Malachite green (MG) were obtained from Shanghai Reagent Co. (Shanghai, China), and these chemicals were analytical reagents and were used without further purification. The solutions were prepared with distilled water without further pH regulation, and all experiments were carried out under room temperature (25 °C) in a water system.

### 3.2. Preparation of TiO_2_

Titanium *n*-butoxide (Ti(OBu)_4_) was employed as the Ti source because the hydrolysis rate of Ti(OBu)_4_ was ca. 150 times slower than that of tetraethyl titanate, Ti(OEt)_4_ [32]. A typical procedure for preparing titania [33] is described as follows: 300 μL of Ti(OBu)_4_ was added into 10 mL of ethanol solution. Then, this newly-formed complex precursor solution was transferred into a 60-mL autoclave containing 5 mL of ultrapure water and was heated at 180 °C for 20 h. The resulting product was collected by centrifugation, washed several times with distilled water and ethanol, respectively, and then dried at 60 °C in a drying oven.

### 3.3. Photocatalytic Experiments

The photocatalytic activities of the TiO_2_ were evaluated by the degradation of MG under the irradiation of a UV lamp (set at 175 W). In a typical process, the TiO_2_ particles were dispersed into an MG solution in a quartz tube under different conditions, including the amount of TiO_2_, pH value and the concentrations of MG. The desired pH of the MG solution was adjusted with 1 M HCl/NaOH to determine the real concentration of MG in the photocatalytic degradation. The solution was then stirred for 2 h in the dark to reach an adsorption–desorption equilibrium between the nanoparticles and the solution. Subsequently, the quartz tube was exposed to irradiation from a UV lamp; 3 mL of MG aqueous solution was intermittently collected at given time intervals for centrifugation, the filtrates measured the absorbance by UV-vis spectroscopy.

### 3.4. Apparatus

The scanning electron microscopy (SEM) images were taken by a Sirion 200 field-emission scanning electron microscope (ThermoFisher, Waltham, MA, USA). X-ray scattering patterns were determined by analyzing the powder samples on a Philips X-Pert Pro X-ray diffractometer (XRD) (Philips, Amsterdam, Holland) with Cu Ka radiation. Transmission electron microscopy (TEM) images were recorded using a JEOL 2010 high resolution transmission electron microscope (Japan Electronics Co., Ltd., Tokyo, Japan), operated at an acceleration voltage of 200 kV. The absorbance of the MG solution was measured using a Lambda 35 UV-vis spectrometer (Perkinelmer, Waltham, MA, USA).

## 4. Conclusions

In summary, TiO_2_ nanoparticles were prepared using a simple and efficient method, which has been proved to be a highly-efficient photocatalyst to degrade MG, a representative and worldwide pollutant in water systems. The kinetics of reaction were successfully monitored by UV spectroscopy, and the kinetic process can be well predicted by the pseudo first-order model. The optimal conditions of the key factors, including TiO_2_ dosage, concentration of MG and pH values, were determined by analyzing the kinetics of the photocatalytic reaction. The maximum efficiency of MG removal was obtained with the conditions of TiO_2_ dosage, pH value and initial concentrations of MG at 0.6 g/L, 8 and 10^−5^ M, respectively. These results provide an efficient strategy to study the photocatalytic degradation of water pollutants.

## Figures and Tables

**Table 1:** Comparison of pseudo first-order model parameters, rate constants (*k*, min^−1^) and *R*^2^ under different TiO_2_ dosages.

TiO_2_ Dosage (g/L)	*k* (min^−1^)	*R* ^2^
0.4	0.0716	0.964
0.6	0.0805	0.995
0.8	0.0711	0.976

**Table 2:** Comparison of pseudo first-order model parameters, rate constants (*k*, min^−1^) and *R*^2^ under different concentrations of MG.

Concentration of MG (M)	*k* (min^−1^)	*R* ^2^
5 × 10^−5^	0.0151	0.965
2.5 × 10^−5^	0.022	0.935
10^−5^	0.0805	0.995
5 × 10^−6^	0.055	0.989

**Table 3:** Comparison of pseudo first-order model parameters, rate constants (*k*, min^−1^) and *R*^2^ under different pH values.

pH Values	*k* (min^−1^)	*R* ^2^
4	0.01	0.961
6	0.039	0.983
8	0.048	0.995
10	0.124	0.737

## Supplementary Materials

The following are available online at http://www.mdpi.com/2079-4991/8/6/428/s1, Figure S1: SEM and TEM imagines of TiO_2_ particles, Figure S2: The UV-vis spectra of TiO_2_, Figure S3: ESR spectral features of the DMPO-·OH spin adducts in the system without addition MG under irradiation of UV light with TiO_2_.
