# Supplementary material for: Optimization of Malachite Green Removal from Water by TiO2 Nanoparticles under UV Irradiation
Source: Nanomaterials (Basel). 2018 Jun 13;8(6):428. doi: 10.3390/nano8060428 (PMC6027246; doi:10.3390/nano8060428)
Supplement: Supplementary file 1 [file nanomaterials-08-00428-s001.pdf]

Optimization of Malachite Green Removal from Water by TiO_2 Nanoparticles under UV Irradiation

Yongmei Ma[1], Maofei Ni[1,2], Siyue Li[1*]

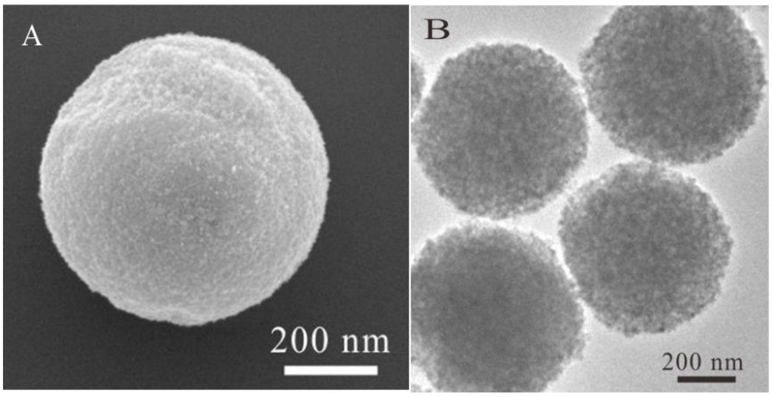

Figure S1. SEM and TEM imagines of TiO_2 particles.

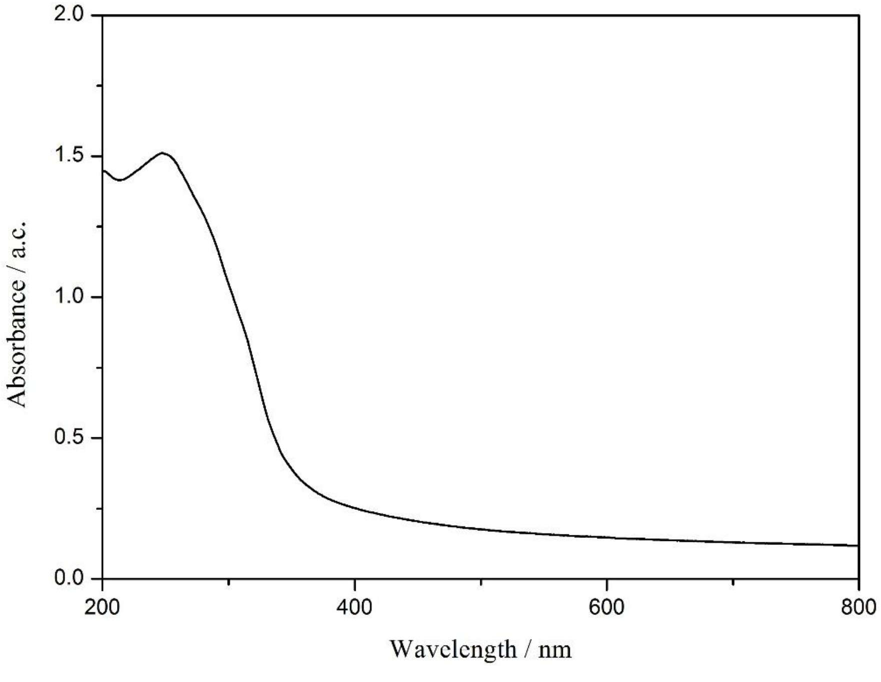

Figure S2. The UV–vis spectra of TiO_2.

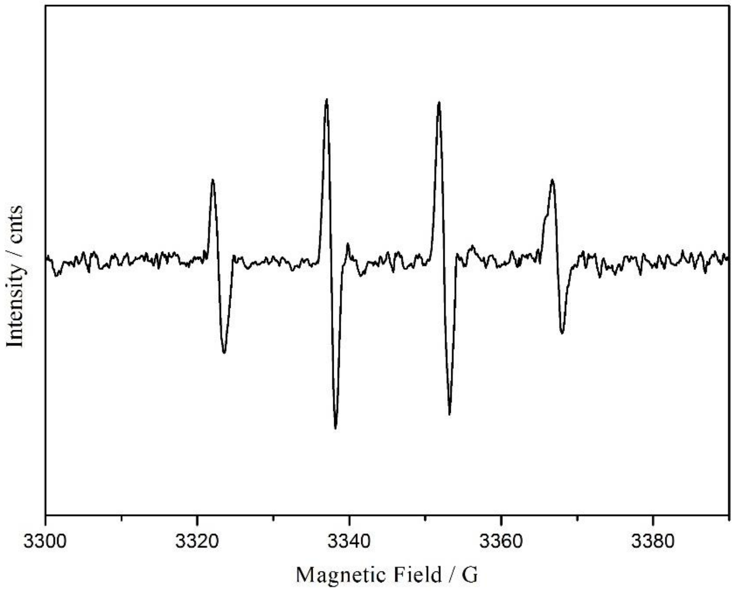

Figure S3. ESR spectral features of the DMPO–·OH spin adducts in the system without addition of MG under irradiation of UV light with TiO_2.
